# Adverse drug reactions and associated patient characteristics in older community-dwelling adults: a 6-year prospective cohort study

**DOI:** 10.3399/BJGP.2022.0181

**Published:** 2023-01-24

**Authors:** Ann S Doherty, Fiona Boland, Frank Moriarty, Tom Fahey, Emma Wallace

**Affiliations:** Health Research Board (HRB) Centre for Primary Care Research, Department of General Practice, Royal College of Surgeons in Ireland (RCSI) University of Medicine and Health Sciences, Dublin.; HRB Centre for Primary Care Research, Department of General Practice, RCSI University of Medicine and Health Sciences, Dublin 2, Ireland; Data Science Centre, RCSI University of Medicine and Health Sciences, Dublin.; School of Pharmacy and Biomolecular Sciences, RCSI University of Medicine and Health Sciences, Dublin.; Health Research Board (HRB) Centre for Primary Care Research, Department of General Practice, Royal College of Surgeons in Ireland (RCSI) University of Medicine and Health Sciences, Dublin.; HRB Centre for Primary Care Research, Department of General Practice, RCSI University of Medicine and Health Sciences, Dublin; Department of General Practice, University College Cork, Cork.

**Keywords:** adverse drug reaction, drug-related side effects and adverse reactions, electronic health records, general practice, older adults, polypharmacy

## Abstract

**Background:**

To date, research on adverse drug reactions (ADRs) has focused on secondary care, and there is a paucity of studies that have prospectively examined ADRs affecting older adults in general practice.

**Aim:**

To examine the cumulative incidence and severity of ADRs and associated patient characteristics in a sample of community-dwelling older adults.

**Design and setting:**

Prospective cohort study of older adults (aged ≥70 years, *N* = 592) recruited from 15 general practices in the Republic of Ireland.

**Method:**

Manual review of the participant’s general practice electronic medical record, linked to the national dispensed prescription medicine database, and a detailed, self-reported patient postal questionnaire. The primary outcomes were ADR occurrence and severity over a 6-year period (2010–2016). Unadjusted and adjusted logistic regression models examined potential associations between patient characteristics and ADR occurrence.

**Results:**

A total of 211 ADRs were recorded for 159 participants, resulting in a cumulative incidence of 26.9% over 6 years. The majority of ADRs detected were mild (89.1%), with the remainder classified as moderate (10.9%). Eight moderate ADRs, representing 34.8% of moderate ADRs and 3.8% of all ADRs, required an emergency hospital admission. ADRs were independently associated with female sex (adjusted odds ratio [OR] 1.83, 95% confidence interval [CI] = 1.17 to 2.85; *P* = 0.008), polypharmacy (5–9 drug classes) (adjusted OR 1.81, 95% CI = 1.17 to 2.82; *P* = 0.008), and major polypharmacy (≥10 drug classes) (adjusted OR = 3.33, 95% CI = 1.62 to 6.85; *P* = 0.001).

**Conclusion:**

This prospective cohort study of ADRs in general practice shows that over one-quarter of older adults experienced an ADR over a 6-year period. Polypharmacy is independently associated with ADR risk in general practice and older adults on ≥10 drug classes should be prioritised for regular medication review.

## INTRODUCTION

As older people are living longer, often with multimorbidity, caring for this population is becoming increasingly complex, and much of this care occurs in primary care.^1^^,^^2^ For the GP, estimating the benefits and harms of a medication in an older person is particularly challenging as comorbidities, concurrent medications, pharmacokinetics, and pharmacodynamics can all impact the clinical outcome.^3^^,^^4^ The prevalence of polypharmacy, defined as ≥5 regular prescribed medications, is increasing, particularly among older people.^5^^,^^6^ Polypharmacy is the primary risk factor for adverse drug reactions (ADRs), one type of medication-related harm.^5^^–^^7^ The World Health Organization (WHO) defines an ADR as any noxious, unintended, and undesired effect of a drug, excluding therapeutic failures, intentional and accidental poisoning, and drug abuse.^8^

Despite limited research to date, ADRs reported incidence in primary care ranges from 6%–80%, reflecting variation in study design, populations, and measurement periods utilised.^9^ Older people are especially vulnerable to ADRs and related adverse outcomes such as emergency admission, drug-related morbidity, and mortality.^4^^,^^10^^–^^13^ ADRs are the cause of nearly 10% of hospitalisations of older adults,^14^ and contribute considerable additional costs to healthcare systems.^15^^,^^16^ A retrospective population cohort study found that ADRs accounted for 9.5% of all direct healthcare costs,^17^ and ADR-related hospitalisations have been estimated to cost the NHS £466 million per annum.^13^

ADRs are heterogeneous by nature, and developing methods to identify those at high risk has proved challenging.^18^ To date, research has focused on secondary care, and there is a paucity of studies that have prospectively examined ADRs in older adults in general practice. In recent systematic reviews of ADRs in primary care (*n* = 33 studies), only two included studies were prospective cohort studies, neither of which were conducted in general practice nor examined older adults specifically.^19^^–^^21^ Furthermore, neither examined ADR prevalence beyond 3 months.^20^^,^^21^ The majority of general practice ADR studies were cross-sectional, with approximately half conducted by screening administrative databases for ADRs recorded during routine care.^19^^,^^22^^–^^24^ Only two studies conducted a medication/medical record review in combination with a patient survey.^25^^,^^26^ This study aimed to examine the cumulative incidence and severity of ADRs and associated patient characteristics in older community-dwelling adults attending general practice.

**Table:** How this fits in

To the authors’ knowledge, no prospective studies have examined adverse drug reaction (ADR) occurrence among older adults attending general practice. In this study, ADRs were found to occur for approximately one in four older adults over a 6-year period. Cardiovascular, nervous system, and anti-infective drugs for systemic use were the most commonly implicated drug classes. Approximately one in four ADRs rated as moderate resulted in additional healthcare utilisation. Female sex, polypharmacy (5–9 drug classes), and major polypharmacy (≥10 drug classes) increased the likelihood of ADRs.

## METHOD

The Strengthening and Reporting of Observational Studies in Epidemiology (STROBE) guidelines were adhered to in the conduct and reporting of this study.^27^ A more detailed description of the methods is presented in Supplementary Appendix S1.

### Study design and population

This is a 6-year (2010–2016) prospective cohort study of older patients (aged ≥70 years) recruited from 15 general practices in Leinster, Republic of Ireland.^28^^,^^29^ A proportionate stratified random sampling approach was used to recruit patients.^30^^,^^31^ Each general practice contributed a number of participants proportionate to the size of the practice. A random sample of patients from each of the 15 participating general practices was invited to take part in the study. The sample was calculated using proportionate stratified random sampling based on the overall sample required, the total number of eligible patients aged ≥70 years in all 15 practices, and assuming a 50% response rate.

Study inclusion criteria were:

- aged ≥70 years on 1 January 2010;
- in receipt of a valid General Medical Services card; and
- in receipt of at least one drug.

Exclusion criteria were:

- receiving palliative care;
- cognitive impairment at the level that would impact ability to complete the outcome measure (defined as Mini-Mental State Examination ≤20);
- significant hearing/speech/visual impairment;
- currently experiencing a psychotic episode;
- hospitalised long-term, in a nursing home, homeless, or in sheltered accommodation; and
- recent (<1 month) bereavement.

Each participant’s GP applied the exclusion criteria at baseline to assess eligibility for participation in this cohort study.

Baseline data collection took place in 2010, with follow-up data collection conducted in 2012 and 2016. Data collection involved review of the participant’s general practice electronic medical record and a detailed, self-reported patient postal questionnaire. Participant consent was obtained to link their medical record and questionnaire data with their prescription dispensing information from the national Health Service Executive-Primary Care Reimbursement Service (HSE-PCRS) database.

**Figure 1.**
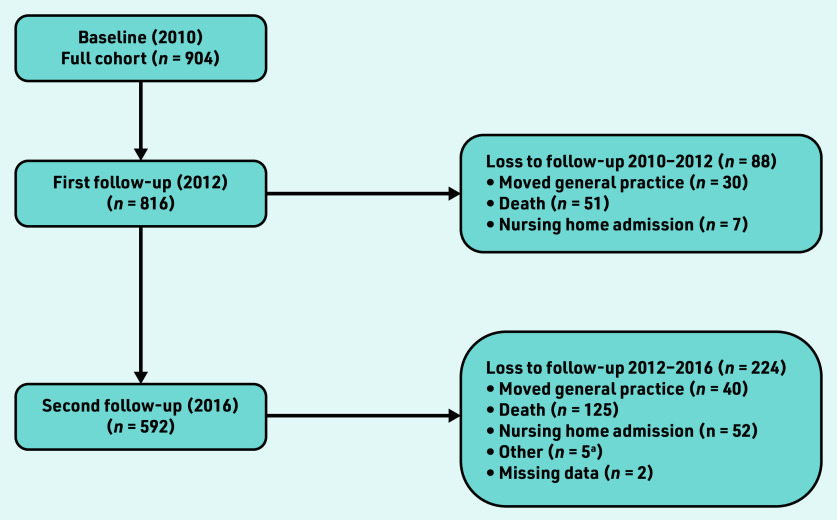
***Study flow diagram (2010–2016) describing losses to follow-up.*** **^a^Hospice, *n* = 2; long-stay inpatient, *n* = 3.**

At baseline, 1487 patients met inclusion criteria and were invited to participate. Of these, 904 participated, representing a response rate of 61%. A total of 592 participants completed three waves of data collection. Losses to follow-up are presented in Figure 1. Descriptive statistics for those who completed study follow-up and those excluded are reported in Supplementary Tables S1–S3. Ethical approval for this study was obtained from the Royal College of Surgeons Ireland University of Medicine and Health Science’s Human Research Ethics Committee.

### Primary outcomes

The primary outcomes were ADR occurrence and severity. ADRs were recorded by manual review of the general practice electronic medical record (see Supplementary Appendix S1). Detecting ADRs using this method involved reviewing each participant’s individual GP consultations and their hospital and other correspondence over the outcome measurement period. Manual chart review, albeit not without its limitations, is considered the gold standard method for the detection of ADRs.^32^^,^^33^ Drug classes were classified using the WHO Anatomical Therapeutic Code (ATC) classification.

ADR causality was assessed using the EU pharmacovigilance working group classification system.^34^ ADR severity was assessed using a previously validated severity classification system: mild, moderate, or severe (see Supplementary Appendix S1).^35^ ADRs were independently rated in terms of severity by an academic pharmacist and an academic GP. Disagreements were resolved by consensus.

### Explanatory variables/patient characteristics

Patient characteristics were selected a priori and recorded at baseline from the general practice electronic medical record (age, sex, deprivation, and multimorbidity according to the Charlson comorbidity index); through linkage to the HSE-PCRS pharmacy claims database (number of drug classes and medication possession ratio [MPR]); and from the patient questionnaire (marital status, private health insurance, and vulnerability using the Vulnerable Elders Survey [VES]-13).^36^ The MPR is a measure of prescription refill and was calculated using the pharmacy claims linked data.^37^ The measure is calculated as the sum of days supplied for all medications (that is, the medication quantity supplied) divided by the time period. The average MPR rate for medication classes, categorised according to the WHO-ATC classification system, was determined for each patient.

### Statistical analysis

Descriptive statistics were utilised to describe the study population and the primary outcomes. The cumulative incidence of ADRs was expressed as the proportion of participants who experienced at least one ADR over the study period (2010–2016). Differences in participant characteristics at baseline were explored. Unadjusted and adjusted logistic regression analyses were used to investigate the association between patient characteristics at baseline and the primary outcome of ADR occurrence. Unadjusted and adjusted odds ratios (ORs), 95% confidence intervals (CIs), and *P*-values were calculated and reported. Stata (version 15) was used for all analyses.

### Sensitivity analysis

A sensitivity analysis was conducted by examining ADR occurrence among participants with at least 2 years of follow-up data (see Supplementary Appendix S1). As the follow-up duration varied for participants in the sensitivity sample, the cumulative incidence of ADRs over 6 years could not be determined. Baseline descriptive statistics are presented for those included in and those excluded from the sensitivity analysis in Supplementary Table S4. For this sample, the proportion of participants who experienced at least one ADR is reported, using the total number of participants in the sensitivity sample (*n* = 816) as the denominator. Both the unadjusted and adjusted logistic regression models controlled for length of follow-up (years).

## RESULTS

### Study population

Baseline descriptive statistics are presented for those with and without the primary outcome of ADR in Table 1 (*n* = 592), and for the full study sample (*n* = 904) in Supplementary Table S1. The median age was 75 years (interquartile range [IQR] 73–79) and 125 (21.1%) participants had multimorbidity (Charlson comorbidity index score ≥2) (see Supplementary Table S1). The median number of drug classes was 5 (IQR 3–7). Overall, 287 (48.5%) participants experienced polypharmacy (5–9 drug classes) and 53 (9.0%) experienced major polypharmacy (≥10 drug classes).

**Table 1.** Descriptive characteristics at baseline for those with and without an ADR over 6 years

**Characteristic**	**Without ADR, *n* = 433, median (IQR)**	**With ADR, *n* = 159, median (IQR)**	***P*-value**
Age, years	75 (73 to 79)	76 (73 to 80)	0.11

Deprivation, patient	1.08 (−0.64 to 2.88)	1.75 (−0.45 to 2.88)	0.18

Number of drug classes	5 (3 to 7)	6 (4 to 8)	<0.001^d^

	***n* (%)**	***n* (%)**	***P*-value**

Sex			
Female	212 (49.0)	110 (69.2)	<0.001^d^
Male	221 (51.0)	49 (30.8)	

Private health insurance	203 (46.9)	73 (45.9)	0.83

Marital status^a^			
Married	226 (52.2)	66 (41.5)	0.03^c^
Separated/divorced	24 (5.5)	6 (3.8)	
Widowed	114 (26.3)	61 (38.4)	
Never married/single	68 (15.7)	26 (16.4)	

Charlson comorbidity index^a^			
0	237 (54.7)	78 (49.1)	0.27
1	103 (23.8)	48 (30.2)	
≥2	92 (21.2)	33 (20.8)	

Medication adherence, MPR ≥80%^b^	273 (63.0)	101 (63.5)	0.54

VES ≥3	112 (25.9)	60 (37.7)	0.005^c^

Polypharmacy			
1–4 drug classes	207 (47.8)	45 (28.3)	<0.001^d^
5–9 drug classes	200 (46.2)	87 (54.7)	
≥10 drug classes	26 (6.0)	27 (17.0)	

a *Missing data (*n*= 1).*

b *Missing data (*n*= 35).*

c P*<0.05.*

d P*<0.001.*

P*-values obtained from Mann–Whitney U test (continuous variables with non-normal distribution) and χ^2^ tests of independence for categorical variables. ADR = adverse drug reaction. IQR = interquartile range. MPR = medication possession ratio. VES = Vulnerable Elders Scale.*

### Primary outcome: ADRs

A total of 211 ADRs were recorded in 159 participants, indicating a cumulative incidence of 26.9% over the 6-year period (2010–2016) (Table 1). Overall, 25 (4.2%) participants experienced two ADRs, seven (1.2%) experienced three ADRs, three (0.5%) experienced four ADRs, and one (0.2%) experienced five ADRs (data not shown). Cardiovascular, nervous system, and anti-infective drugs were most commonly implicated in ADRs (Table 2). Regarding ADR severity, 188 (89.1%) ADRs were classified as mild and 23 (10.9%) as moderate, with no ADRs categorised as severe (data not shown). A total of 10 moderate ADRs (4.7% of all ADRs) resulted in additional healthcare utilisation. Two ADRs resulted in an outpatient appointment and eight ADRs in emergency hospital admission. Thus, 34.8% of moderate ADRs (representing 3.8% of all ADRs) resulted in an emergency admission. No ADRs resulting in death were detected. Examples of the different types of ADRs experienced by degree of severity are presented in Box 1.

**Box 1.** Examples of ADRs by severity

**ADR Severity**	**ADR details**	**WHO-ATC code(s)**
Mild	Gastrointestinal upset; nausea; vomiting; constipation; and diarrhoea	A02, A10, B03, C03, C09, G04, H05, J01, M01, M05, N01, N02, N06
	Headaches	A02, C01, C08, N02, N03, R06
	Dry mouth	C03, G04, N02, N06
	Dizziness	A10, C02, C03, C07, C08, C09, N02, N06
	Sedation	N02, N03, N06, N07, R06
	Oedema	C03, C08, M01

Moderate	Gastrointestinal upset, resulting in hospitalisation	B01
	Confusion, hallucinations	N02
	Hyponatraemia	N06
	Dystonic reaction	N05
	High INR, resulting in hospitalisation	B01

*ADR = adverse drug reaction. INR = international normalised ratio. WHO-ATC = World Health Organization Anatomical Therapeutic Code.*

**Table 2.** Drug classes implicated in ADRs according to the WHO-ATC classification system (*n* = 159 study participants)

**WHO-ATC class**		**ADRs, *n***	**% all ADRs**
**A**	**Alimentary tract and metabolism**	**18**	**8.53**
A02	Drugs for acid-related disorders	10	4.74
A07	Antidiarrhoeal, intestinal anti-inflammatory/anti-infective agents	1	0.47
A10	Drugs used in diabetes	5	2.37
A11	Vitamins	1	0.47
A12	Mineral supplements	1	0.47

**B**	**Blood and blood forming organs**	**4**	**1.90**
B01	Antithrombotic agents	3	1.42
B03	Antianaemic preparations	1	0.47

**C**	**Cardiovascular system**	**69**	**32.70**
C01	Cardiac therapy	5	2.37
C02	Antihypertensives	2	0.95
C03	Diuretics	13	6.16
C07	Beta blocking agents	4	1.90
C08	Calcium channel blockers	17	8.06
C09	Agents acting on the renin-angiotensin system	19	9.00
C10	Lipid-modifying agents	9	4.27

**G**	**Genito-urinary system and sex hormones**	**10**	**4.74**
G04	Urologicals	10	4.74

**H**	**Systemic hormonal preparations, excluding sex hormones and insulins**	**4**	**1.90**
H03	Thyroid therapy	3	1.42
H05	Calcium homeostasis	1	0.47

**J**	**Anti-infectives for systemic use**	**26**	**12.32**
J01	Antibacterials for systemic use	26	12.32

**L**	**Antineoplastic and immunomodulating agents**	**1**	**0.47**
L04	Immunosuppressants	1	0.47

**M**	**Musculoskeletal system**	**14**	**6.64**
M01	Anti-inflammatory and antirheumatic products	10	4.74
M05	Drugs for treatment of bone diseases	4	1.90

**N**	**Nervous system**	**61**	**28.91**
N01	Anaesthetics	4	1.90
N02	Analgesics	28	13.27
N03	Antiepileptics	7	3.32
N04	Anti-Parkinson’s drugs	1	0.47
N05	Psycholeptics	4	1.90
N06	Psychoanaleptics	16	7.58
N07	Other nervous system drugs	1	0.47

**R**	**Respiratory system**	**4**	**1.90**
R03	Drugs for obstructive airway diseases	2	0.95
R06	Cough and cold preparations	2	0.95

*ADR = adverse drug reaction. WHO-ATC = World Health Organization Anatomical Therapeutic Code.*

### Associations between patient characteristics and ADRs

Unadjusted associations (Table 3) for ADRs were observed for female sex, marital status, VES-13 score, polypharmacy (5–9 drug classes), and major polypharmacy (≥10 drug classes). In the adjusted model, independent associations remained for female sex (OR 1.83, 95% CI = 1.17 to 2.85, *P* = 0.008), with a dose-response relationship observed for polypharmacy (5–9 drug classes) (OR 1.81, 95% CI = 1.17 to 2.82, *P* = 0.008), and major polypharmacy (≥10 drug classes) (OR 3.33, 95% CI = 1.62 to 6.85, *P* = 0.001).

**Table 3.** Unadjusted (*n*= 592) and adjusted (*n* = 555) logistic regression for at least one ADR over 6 years (2010–2016)

**Characteristic**	**Unadjusted**		**Adjusted**	

	**OR (95% CI)**	***P*-value**	**OR (95% CI)**	***P*-value**
Age	1.04 (0.99 to 1.08)	0.09	1.00 (0.95 to 1.05)	0.87

Deprivation	1.05 (0.98 to 1.13)	0.17	1.07 (0.98 to 1.16)	0.15

Sex, female	2.34 (1.59 to 3.44)	<0.001^a^	1.83 (1.17 to 2.85)	0.008^b^

Private health insurance	0.96 (0.67 to 1.38)	0.83	1.33 (0.86 to 2.07)	0.20

Medication adherence, MPR ≥80% ^c^	0.88 (0.60 to 1.31)	0.54	0.81 (0.53 to 1.22)	0.31

Marital status^d^		0.03^b^		
Separated/divorced	0.86 (0.34 to 2.18)	0.75	0.76 (0.29 to 1.99)	0.57
Widowed	1.83 (1.21 to 2.77)	0.004^b^	1.36 (0.84 to 2.20)	0.22
Never married/single	1.31 (0.77 to 2.22)	0.32	1.21 (0.67 to 2.17)	0.52

Charlson comorbidity^e^		0.28		
1	1.42 (0.92 to 2.17)	0.11	1.22 (0.76 to 1.95)	0.41
≥2	1.09 (0.68 to 1.75)	0.72	0.97 (0.57 to 1.65)	0.90

VES ≥3	1.74 (1.18 to 2.56)	0.005^b^	1.22 (0.77 to 1.96)	0.40

Polypharmacy^f^		<0.001^a^		
5–9 drug classes	2.00 (1.33 to 3.01)	0.001^b^	1.81 (1.17 to 2.82)	0.008^b^
≥10 drug classes	4.78 (2.55 to 8.95)	<0.001^a^	3.33 (1.62 to 6.85)	0.001^b^

a *P<0.001.*

b *P<0.05.*

c *Missing data (*n *= 35 cases).*

d *Referent married, missing data (*n *= 1 case).*

e *Referent 0, missing data (*n *= 1 case).*

f *Referent 0–4 drug classes. ADR = adverse drug reaction. MPR = medication possession ratio. OR = odds ratio. VES = Vulnerable Elders Scale.*

### Sensitivity analysis

A sensitivity analysis examined ADR occurrence for participants with at least 2 years of follow-up data (*n* = 816). Baseline descriptive statistics are presented for those with and without the primary outcome of ADR in Supplementary Table S5. A total of 259 ADRs relating to 199 participants were included; thus, 24.4% of participants experienced at least one ADR. In the adjusted model, female sex (OR 1.68, 95% CI = 1.14 to 2.47, *P* = 0.009), deprivation (OR 1.09, 95% CI = 1.01 to 1.17, *P* = 0.03), polypharmacy (5–9 drug classes: OR 1.87; 95% CI = 1.24 to 2.82, *P* = 0.003) and major polypharmacy (≥10 drug classes: OR 2.72, 95% CI = 1.50 to 4.93, *P* = 0.001) were associated with an increased likelihood of experiencing an ADR (see SupplementaryTable S6).

## DISCUSSION

### Summary

This prospective cohort study over 6 years shows that ADRs are common in older people attending general practice, with approximately one in four (26.9%) experiencing at least one ADR over the period. While the majority of ADRs are mild (89.1%), approximately one-third (34.8%) of moderate ADRs result in an emergency admission. In total, 10 ADRs (4.7%) resulted in additional healthcare utilisation (outpatient appointment or hospitalisation). Drug classes most commonly implicated include: cardiovascular system drugs (for example, amlodipine and furosemide), nervous system drugs (for example, citalopram, mirtazapine, and tramadol), and anti-infectives for systemic use (for example, amoxicillin and co-amoxiclav). ADRs were independently associated with female sex, polypharmacy (5–9 drug classes), and major polypharmacy (≥10 drug classes), while the likelihood for ADR increased more than threefold for those with major polypharmacy.

### Strengths and limitations

Strengths of this study include the manual review of general practice electronic medical records, considered the gold standard for ADR detection.^32^ Previous reviews of ADRs in general practice have reported a small number of studies, with inconsistent methodology.^9^^,^^19^ This study extends the evidence base by reporting ADR cumulative incidence and severity over 6 years. Furthermore, the data collected allowed for the inclusion of several confounding variables (for example, multimorbidity, medication adherence, and functional status) in the statistical analysis. The robustness of the study findings is supported by the sensitivity analysis. In terms of study limitations, ADRs (mild, moderate, or severe) could have accounted for admission to a care home and/or death among this older cohort. Over the course of 6 years of follow-up, death occurred in 176 (19.5%) participants, while 59 (6.5%) were admitted to a care home. Caution is required in interpreting overall incidence of ADRs for this reason. A recent retrospective analysis of VigiBase, the WHO’s pharmacovigilance database, investigated fatal adult ADRs (2010–2019) reported by physicians.^38^ Of >3.2 million included ADRs, just over 1% were fatal, with males, patients aged >65 years, and those taking antineoplastic/immunomodulating drugs at higher risk. There was also significant variation in fatal ADR rates across different countries and continents. Future studies need to obtain ADR data when patients die or transition to care home settings*.* Another limitation to this study is that it was not possible to classify ADRs by type or preventability, nor was it possible to look at ADR annual incidence. Lastly, recruitment is limited to a regional health area and potentially limits the generalisability of the study findings.

### Comparison with existing literature

Understanding of the impact of ADRs in general practice has been limited by the lack of research conducted to date. A systematic review by Insani *et al* of 33 primary care studies reported 10 general practice studies (nine cross-sectional and one retrospective cohort study).^19^ Only two general practice studies used medical record and patient survey methodology, whereas five studies screened administrative databases for ADRs recorded during routine care. Furthermore, only two studies examined ADRs in older adults specifically.^26^^,^^39^ Two prospective studies have been conducted in primary care internal medicine settings; however, neither examined ADR occurrence beyond 3 months, nor examined older adults specifically.^20^^,^^21^ To the best of the authors’ knowledge, this is the first prospective general practice study examining ADRs among older adults.

The systematic review by Insani *et al* (total population >1.5 million participants) further reported a pooled ADR prevalence rate of 8.32%.^19^ Notably, this pooled estimate is predicated mostly on cross-sectional studies and also includes paediatric populations. Subgroup analysis found prevalence estimates varied according to age, ADR detection method, setting, and sample size. In the present study, the cumulative incidence of ADRs over 6 years (26.9%) is congruent with the pooled prevalence of ADRs among those aged ≥65 years (28.43%) identified in their subgroup analysis.^19^

The majority of ADRs detected were rated as mild (89.1%), with the remainder (10.9%) rated as moderate. Several primary care studies report the proportion of mild ADRs to range from 2.2%–45.9% and moderate ADRs to range from 42.2%–96.4%.^40^^–^^42^ Ten ADRs (4.7%) resulted in either an outpatient appointment or hospital admission, which is comparable to the 1.3%–9.1% of ADRs reported to require an emergency department visit and/or hospital admission.^19^

The most commonly identified drug classes (diuretics, calcium channel blockers, angiotensin-converting enzyme inhibitors, opioid analgesics, and antidepressants) are consistent with higher-risk drug classes reported previously and represent those most commonly prescribed in primary care.^9^^,^^19^^,^^20^^,^^22^^,^^42^^–^^45^ Two general practice studies found that cardiovascular drugs were implicated in approximately 18% of ADRs.^22^^,^^42^ In the present study, cardiovascular drugs were implicated in 32.7% of all ADRs, which is comparable to rates identified in primary care internal medicine settings (23.7%–31.0%).^20^^,^^44^ A sizeable proportion of ADRs (28.9%) were attributed to nervous system drugs. The systematic review by Insani *et al* reported a median ADR rate of 13.4% for nervous system drugs across eight studies (range: 3.5%–39.6%).^19^

Female sex and polypharmacy (both 5–9 drug classes and ≥10 drug classes) are associated with experiencing an ADR in multivariable analyses, which aligns with findings consistently reported in the literature.^4^^,^^20^^,^^23^^,^^46^^–^^49^ Polypharmacy may serve as a modifiable target for reducing ADR risk in the context of deprescribing initiatives for potentially inappropriate medications and those no longer clinically indicated.

### Implications for research and practice

The findings may inform future initiatives, including structured medication reviews (SMRs) in general practice, by highlighting several intervention targets. Cardiovascular, nervous system, and anti-infective drugs were identified as the higher-risk drug classes and represent the most commonly prescribed medications in general practice.^45^ Through a shared decision-making approach, GPs and their patients need to balance the benefits and risks of these agents. The potential for ADRs, which are often difficult to diagnose in older adults,^4^^,^^50^ should form part of every differential diagnosis for older patients, especially those who have recently started a new medication or experienced a dose adjustment. ADRs can be difficult to identify in medically complex older adults as they often present as non-specific symptoms such as delirium, drowsiness, falls, fatigue, and constipation, all of which have several potential causes.^4^^,^^47^ ADR symptoms may be mistaken as the onset of a new medical problem or related to an existing diagnosis, rather than being secondary to medication.^47^ The failure to recognise an ADR may result in a prescribing cascade, where a new medication is initiated to treat the ADR, thereby exposing the older adult to additional risk.^51^^,^^52^

The findings also indicate that the greater the medication burden the greater the likelihood for medication-related harm. Those prescribed ≥10 drug classes were over three times more likely to experience an ADR, and therefore may receive the optimum benefit from SMRs. Existing guidance, such as the Scottish Polypharmacy Guidance, has emphasised the prioritisation of SMRs for those prescribed high-risk drug classes and ≥10 medications.^51^ The findings provide additional evidence to support such guidance where case finding indicators have previously been developed by clinical consensus. From a policy perspective, SMRs have been identified as a strategic intervention to address the estimated 10% overprescribing of medications in primary care.^52^^,^^53^ This study shows that addressing polypharmacy is a critical component in reducing medication burden and lessening the likelihood of ADRs for vulnerable patients.

In conclusion, ADRs are common among older adults in general practice, with females and those with major polypharmacy at highest risk. While the majority of ADRs identified were mild, a considerable proportion of moderate ADRs resulted in additional healthcare utilisation. ADRs can be difficult to identify in medically complex older adults as they often present as non-specific symptoms. GPs are well placed to detect the occurrence of ADRs from drugs prescribed in primary care as well as in other care settings.^54^ Deprescribing of ineffective medications and those no longer clinically indicated is one approach to reducing the risk of ADRs in older patients.

## Data

The dataset analysed during the current study is not publicly available in accordance with the consent provided by participants. The participants of this study did not give written consent for their data to be shared publicly. Data may be made available following a reasonable request and ethical approval.
